# Potent Bioactive Compounds From Seaweed Waste to Combat Cancer Through Bioinformatics Investigation

**DOI:** 10.3389/fnut.2022.889276

**Published:** 2022-04-22

**Authors:** Kaushik Kumar Bharadwaj, Iqrar Ahmad, Siddhartha Pati, Arabinda Ghosh, Tanmay Sarkar, Bijuli Rabha, Harun Patel, Debabrat Baishya, Hisham Atan Edinur, Zulhisyam Abdul Kari, Muhammad Rajaei Ahmad Mohd Zain, Wan Ishak Wan Rosli

**Affiliations:** ^1^Department of Bioengineering and Technology, Gauhati University, Guwahati, India; ^2^Division of Computer Aided Drug Design, Department of Pharmaceutical Chemistry, R. C. Patel Institute of Pharmaceutical Education and Research, Shirpur, India; ^3^NatNov Bioscience Private Limited, Balasore, India; ^4^Skills Innovation & Academic Network (SIAN) Institute-Association for Biodiversity Conservation and Research, Balasore, India; ^5^Microbiology Division, Department of Botany, Gauhati University, Guwahati, India; ^6^Department of Food Processing Technology, Malda Polytechnic, West Bengal State Council of Technical Education, Govt. of West Bengal, Malda, India; ^7^School of Health Sciences, Universiti Sains Malaysia, Kubang Kerian, Malaysia; ^8^Department of Agricultural Sciences, Faculty of Agro-Based Industry, Universiti Malaysia Kelantan, Kelantan, Malaysia; ^9^Department of Orthopaedics, School of Medical Sciences, Universiti Sains Malaysia, Kubang, Malaysia; ^10^Nutrition Programme, School of Health Sciences, Universiti Sains Malaysia, Kubang Kerian, Malaysia

**Keywords:** HDAC 2, seaweed, molecular docking, molecular dynamics simulation, MM-GBSA

## Abstract

The seaweed industries generate considerable amounts of waste that must be appropriately managed. This biomass from marine waste is a rich source of high-value bioactive compounds. Thus, this waste can be adequately utilized by recovering the compounds for therapeutic purposes. Histone deacetylases (HDACs) are key epigenetic regulators established as one of the most promising targets for cancer chemotherapy. In the present study, our objective is to find the HDAC 2 inhibitor. We performed top-down *in silico* methodologies to identify potential HDAC 2 inhibitors by screening compounds from edible seaweed waste. One hundred ninety-three (*n* = 193) compounds from edible seaweeds were initially screened and filtered with drug-likeness properties using SwissADME. After that, the filtered compounds were followed to further evaluate their binding potential with HDAC 2 protein by using Glide high throughput virtual screening (HTVS), standard precision (SP), extra precision (XP), and quantum polarized ligand docking (QPLD). One compound with higher negative binding energy was selected, and to validate the binding mode and stability of the complex, molecular dynamics (MD) simulations using Desmond were performed. The complex-binding free energy calculation was performed using molecular mechanics-generalized born surface area (MM-GBSA) calculation. Post-MD simulation analyses such as PCA, DCCM, and free energy landscape were also evaluated. The quantum mechanical and electronic properties of the potential bioactive compounds were assessed using the density functional theory (DFT) study. These findings support the use of marine resources like edible seaweed waste for cancer drug development by using its bioactive compounds. The obtained results encourage further *in vitro* and *in vivo* research. Our *in silico* findings show that the compound has a high binding affinity for the catalytic site of the HDAC 2 protein and has drug-likeness properties, and can be utilized in drug development against cancer.

## Introduction

After terrestrial plants and microorganisms, marine organisms are considered to be a prominent source of drug discovery motivation. The massive diversification of organisms in the marine ecosystem serves as a reservoir of a wide range of natural compounds (1, 2). New marine molecules and their biological potential are discovered every year, allowing these compounds to be used in functional foods and the development of new nutraceuticals and therapeutic drugs (3, 4). In Asian countries, particularly China, Korea, and Japan, marine algae have long been consumed as food, whereas in Western countries, algae are mostly used as a source of food additives (5), natural pigment (6), and minerals (7). The consumption of edible algae, as well as the establishment of algae-based companies, has recently dramatically developed (8). Macroalgae or seaweeds and microalgae are the two types of dominant photosynthetic aquatic organisms. Seaweeds (Marine algae) are a type of photosynthetic non-flowering plant-like multicellular algae found in the ocean that are rich in proteins, vitamins, minerals, and a vast number of bioactive compounds possessing significant antibacterial, anticancer, antifungal, and antiviral properties (9, 10). Around 6,000 species of seaweeds are found in deep oceans, up to 180 m deep. They are sometimes seen in shallow coastal waters. Seaweeds are classified into three major categories based on their pigmentation: red (Rhodophyta), green (Chlorophyta), and brown (Phaeophyceae; phylum: Ochrophyta) algae (11). Sulfated polysaccharides and bioactive compounds are abundant in algae that grow in saline seas or near river mouths, transforming seaweeds into potential pharmaceutical agents (12).

The increasing demand and growth in seaweed farming for seaweed consumables encourages producers to enhance their degree of seaweed processing and cultivation (13). This situation leads to an uncontrolled and uneven quality of the seaweeds' nutritive composition and pharmacological characteristics. In turn, it results in large amounts of seaweed waste usually being dumped in landfills as garbage. Industrial rejection, uneven harvest time, drying processes, and packaging waste are scattered at the processing site (14, 15). Thus, the seaweed waste can be collected and can be potentially applied in both the food and pharmaceutical industries. It has already been established and widely known that diverse groups of seaweeds contain different bioactive compounds possessing a wide range of pharmacological properties. Thus, the seaweed waste can be collected and used in pharmaceutical industries. There needs to be a way to use this potential seaweed debris for sustainable development.

Cancer is a severe issue that threatens the health of all human communities (16), and natural products have played an important role in the history of anticancer medication development (17–20). A large number of experiments in recent years have shown potent anti-cancer properties of several seaweed-derived compounds by inhibitions of tumor development, adhesion, invasion, and metastasis (10, 21, 22). Also, in recent years, much emphasis has been placed on exploring the anti-proliferative activity of bioactive compounds from edible seaweed. With this in mind, we investigate the anticancer properties of compounds from some species of edible seaweed. The phloroglucinol compound isolated from *Ecklonia cava* has good anti-proliferative activity in human breast cancer cells (MCF 7). Similarly, extracts from the brown algae *Laminaria japonica* inhibit human hepatocellular carcinoma (BEL7402) and murine leukemic cells from proliferating (P388). Fucoidan derived from the brown algae has shown anti-cancer activity in human colon cancer cells (HCT116) by activating the apoptosis signals of cancer cells and thereby inducing apoptosis. Furthermore, several seaweed compounds have been tested against different types of glioblastoma cells with promising results in suppressing cell survival while causing no adverse effects on the cells (23, 24).

Histone modifications, specifically histone acetylation/deacetylation, are an important epigenetic regulatory system that is engaged in a variety of human cancers. Histone deacetylation is a key regulator of gene transcription that involves the removal of acetyl groups. Histone deacetylases are a family of enzymes that regulate this mechanism (HDACs). HDACs are categorized into four categories: class I (HDAC 1, 2, 3, and 8), class II (IIa: HDAC 4, 5, 7, and 9; IIb: HDAC 6 and 10), class III (SIRT1, 2, 3, 4, 5, 6, and 7), and class IV (SIRT1, 2, 3, 4, 5, 6, and 7; HDAC11). HDAC dysregulation is correlated to a poor prognosis in a variety of human cancers, making these enzymes a promising therapeutic target (25, 26). HDAC inhibition has been shown to induce cell cycle arrest, tumor angiogenesis inhibition, differentiation of some transformed cell lines, and/or apoptosis in tumor cells, indicating its potential as a therapeutic target for cancer treatment (27). HDAC 2 overexpression plays a significant role in cancer progression. For example, deregulation of p53, an important tumor suppressor protein, is frequently found in tumors, and HDAC 2 deacetylation is one of the reasons, indicating its significance in specificity. Thus, developing a potent HDAC 2 selective inhibitor was useful for a potent therapeutic strategy for cancer progression and development (28).

This study implemented top–down and structure-based high-throughput screening methodologies to find novel HDAC 2 inhibitors from marine edible seaweed compounds by using the comprehensive marine natural products database (CMNPD). CMNPD is a free online database containing marine natural products (MNPs) from the ocean to facilitate drug discovery. Thousands of compounds with various physicochemical and pharmacokinetic properties, biological activity data, taxonomy, and geographic distribution of source organisms are contained in the CMNPD database. We focused on marine edible seaweed compounds to investigate novel HDAC 2 inhibitors as potential anticancer compounds. We used various *in silico* methods such as drug-likeness prediction, molecular docking, molecular dynamics simulation, MM-GBSA, free energy landscape, PCA, DCCM, ADME calculation, and DFT study.

## Materials and Methods

### Preparation of Ligands and Receptors

A total of 17 edible seaweed species were selected for our investigation (Table 1). Out of this, 193 edible seaweed compounds from the Comprehensive Marine Natural Products Database (CMNPD) (https://www.cmnpd.org) were used in this study. The Canonical SMILE format of the compounds from the database (Supplementary File S1) was retrieved, the structure was built using the structure builder option of Chimera, and geometry optimization was performed by using freely available UCSF Chimera version 1.14 software (29) before conducting experiments. The obtained structures in 3-D format were saved as.pdb files.

**Table 1:** List of edible seaweed studied for identification of prospective HDAC 2 inhibitors to combat cancer.

**Sl. no**.	**Seaweed name**	**Number of compounds studied**
1.	*Halidrys siliquosa*	10
2.	*Saccharina japonica*	9
3.	*Ascophyllum nodosum*	2
4.	*Sargassum fulvellum*	1
5.	*Undaria pinnatifida*	3
6.	*Sargassum fusiforme*	4
7.	*Ecklonia cava*	1
8.	*Osmundea pinnatifida*	24
9.	*Ulva lactuca*	24
10.	*Rhodomela confervoides*	46
11.	*Leathesia marina*	8
12.	*Eisenia bicyclis*	10
13.	*Vertebrata lanosa*	5
14.	*Neorhodomela larix*	2
15.	*Ecklonia stolonifera*	7
16.	*Caulerpa racemosa*	35
17.	*Pyropia yezoensis*	2

The crystal structure of HDAC 2 (PDB ID: 4LY1) protein was obtained from the protein data bank (PDB) repository (www.rcsb.org) with a resolution of 1.57 Å. This structure was selected from the protein data bank due to a higher resolution than the other structures in the database. The PDB file of HDAC 2 structure was processed and refined with the Protein preparation wizard [Version 2021-2, Schrodinger (30, 31)]. Ions, cofactors, and water molecules were deleted and hydrogens were added to the heavy atoms. Selenomethionines were converted to methionines. Het states were generated using Epik at pH 7.0 ± 2.0. OPLS_2005 force field (32) was used for protein minimization.

### Drug-Likeliness and PAINS Filtering

SwissADME (http://www.swissadme.ch/) was used to screen potential bioactive compounds from edible seaweed by calculating the drug-likeness properties that constitute Lipinski (33, 34), Ghose (35), Veber (36), and PAINS (37) as per our previous study (38).

### Molecular Docking Profiling of Seaweed Compounds

A grid was generated by taking into account the co-crystal ligand that was found in the active site of the selected protein target HDAC 2 (PDB ID: 4LY1). A grid box was generated (*x* = 22.19; *y* = −18.4; *z* = 0.63) at the centroid of the active site. The compounds were sequentially docked into the catalytic pocket of HDAC 2 (PDB ID: 4LY1). The glide program and its virtual screening workflow (VSW) process, including three docking protocols, namely high throughput virtual screening (HTVS), standard precision (SP) module, and extra precision (XP) module, were applied. Each ligand was docked to the crystal structure of HDAC 2 using the HTVS, resulting in one pose. Though the SP docking protocol provides a good scoring function retaining the good scoring states, about 10% of the total edible seaweed compounds were shifted from HTVS to SP, which aids in the detection of false-positive results. Moreover, about 10% of SPs total edible seaweed compounds were processed to XP, where XP provides the best scoring states (39).

### Quantum Polarized Ligand Docking (QPLD)

In protein–ligand molecular docking, QPLD was used to determine the right electrical charges. QPLD is a docking method that analyzes ligand/protein interactions by combining the QM and the MM approaches. The interaction energy of the protein/newly produced ligand pose was estimated using the QSite programe in Schrödinger, with the protein being treated with the molecular mechanical (MM) approach and the ligand pose being treated with the quantum mechanical (QM) method (40). For the ligand pose within the protein environment, the Qsite algorithm created a new set of atomic partial charges. Using the Glide software with the XP-scoring function, the ligand configuration with QM-generated partial charges was re-docked to the protein (41). The same grid file that was generated by the Glide grid generating tool was utilized for QPLD, and the same ligand was selected in the ligand to be the docked option.

### MD Simulations and Free Energy Landscape Analysis

The molecular dynamics simulations of the docked complex of HDAC 2 protein with the selected edible seaweed compound, cinnamyl dihydrocinnamate, were carried out using the Desmond 2020.1 from Schrödinger, LLC (42) to envisage the dynamic behavior, intermolecular interaction, and stability of the docked complex (43–45). The complex was prepared using the system builder platform by solvation with the simple point-charge (SPC) explicit water model in the orthorhombic simulation box. The solvated complex system was neutralized with a suitable number of Na^+^/Cl^−^ counter ions and a salt concentration of 0.15 M to mimic the physiological conditions (46). The receptor–ligand complex system was designated with the OPLS-2005 force field, and an explicit solvent model with the SPC water molecules was used in this system (47–51). The HDAC 2 (protein)-cinnamyl dihydrocinnamate (ligand) complex system was initially equilibrated using an NVT ensemble for 100 ns. Following the previous phase, a short run of equilibration and minimization was carried out using an NPT ensemble for 12 ns. The NPT ensemble was set up using the Nose-Hoover chain coupling scheme (52, 53) with a temperature of 37°C, a relaxation duration of 1.0 ps, and a pressure of 1 bar. A time step of 2 fs was chosen. With a relaxation time of 2 ps, the Martyna–Tuckerman–Klein chain coupling system (54) barostat method was used for pressure control. The particle mesh Ewald technique (55) was used to calculate long-range electrostatic interactions, with the radius for Coulomb interactions set at 9Å (55). The bonded forces were calculated utilizing the RESPA integrator with a time step of 2 fs for each trajectory. Finally, to monitor the binding stability of the protein–ligand docked complex, the root mean square deviation (RMSD), the radius of gyration (Rg), root mean square fluctuation (RMSF), the number of hydrogen (H-bonds), plot of ligand interaction in the binding cavity, stacked bar chart plot of protein–ligand contact analysis, total contacts timeline analysis of MD trajectory, and analysis of the torsional degree of freedom for the rotatable bonds of the selected ligand were calculated by the results obtained after MD simulations of protein–ligand docked complex of 100 ns of simulation time.

The free energy landscape of protein folding on HDAC 2 protein–cinnamyl dihydrocinnamate complex was measured using geo_measures v 0.8 (56). The MD trajectory vs. RMSD and radius of gyration (Rg) energy profile of protein folding was recorded in a 3-dimensional plot using the matplotlib python package utilizing Geo_measures, which includes a sophisticated library of g_sham.

### Molecular Mechanics-Generalized Born Surface Area (MM-GBSA) Calculations

The binding free energies of the HDAC 2 protein (receptor) and ligands *viz*., cinnamyl dihydrocinnamate complex were investigated using the molecular mechanics-generalized born surface area (MM-GBSA) methodology using the prime module's Python script *thermal mmgbsa.py* (57, 58). OPLS 2005 force field, VSGB solvent model, and rotamer search methods were used to calculate the binding free energy (59). The binding free energy calculation upon binding of ligands with the receptor HDAC 2 was calculated using the following equations:


(1) $$\begin{array}{l} \Delta G_{\text{bind}}=G_{\text{complex}}-\left(G_{\text{protein}}+G_{\text{ligand}}\right) \end{array}$$

where ΔG_bind_ = binding free energy, G_complex_ = free energy of the complex, G_protein_ = free energy of the target protein, and G_ligand_ = free energy of the ligand.

### Principal Component Analysis (PCA) and Dynamic Cross-Correlation Matrix (DCCM) of the MD Simulation Trajectories

Principal component analysis analysis was performed to recover the global movements of the trajectories during a 100-ns simulation of cinnamyl dihydrocinnamate complex with HDAC 2. A covariance matrix was generated to calculate the PCA. The movements of trajectories of 10 alternative conformational modes of the main component were calculated for conformational analysis of the cinnamyl dihydrocinnamate in a complex bound with HDAC 2 protein, and a comparison of the first highest mode (PC 2) was investigated. To investigate domain correlations, the dynamic cross-correlation matrix (DCCM) was built across all Cα-atoms during a 100-ns MD simulation for HDAC 2-compound bound complex. The DCCM was carried out using Schrodinger's trj essential dynamics script (30).

### Density Functional Theory (DFT) Calculations

The Jaguar module in Schrödinger was used to perform DFT calculations. The basis set 6-31G^*^ was used to do a complete geometry optimization using the Hybrid DFT model with Becke's three-parameter exchange potential and Lee-Yang-Parr's correlation functional (B3LYP) technique. DFT energetics depicts the 3D electronic states of molecules to assess the transfer of electrons and reactivity in a specific environment. The quantum chemical properties of the promising compound, like molecular orbital (HOMO), the lowest unoccupied molecular orbital (LUMO), and the energy gap were calculated along with the molecular electrostatic potential surface [MESP; (60, 61)].

### *In silico* ADMET Property Prediction of the Compound

Using the Schrodinger suite's QikProp module, the absorption, distribution, metabolism, and excretion (ADME) properties of the chosen compound were determined (62, 63). SASA, FOSA, FISA, PISA, WPSA, QPlogPo/w, QPlogS, QPlogkhsa, QplogBB, QPPCaco percent human oral absorption, rule of five, and rule of three were compared to a manual-recommended range of values for ADME attributes (62). QikProp's descriptors were used to make ADMET predictions and calculate drug-likeness parameter calculations.

**Figure 1:**
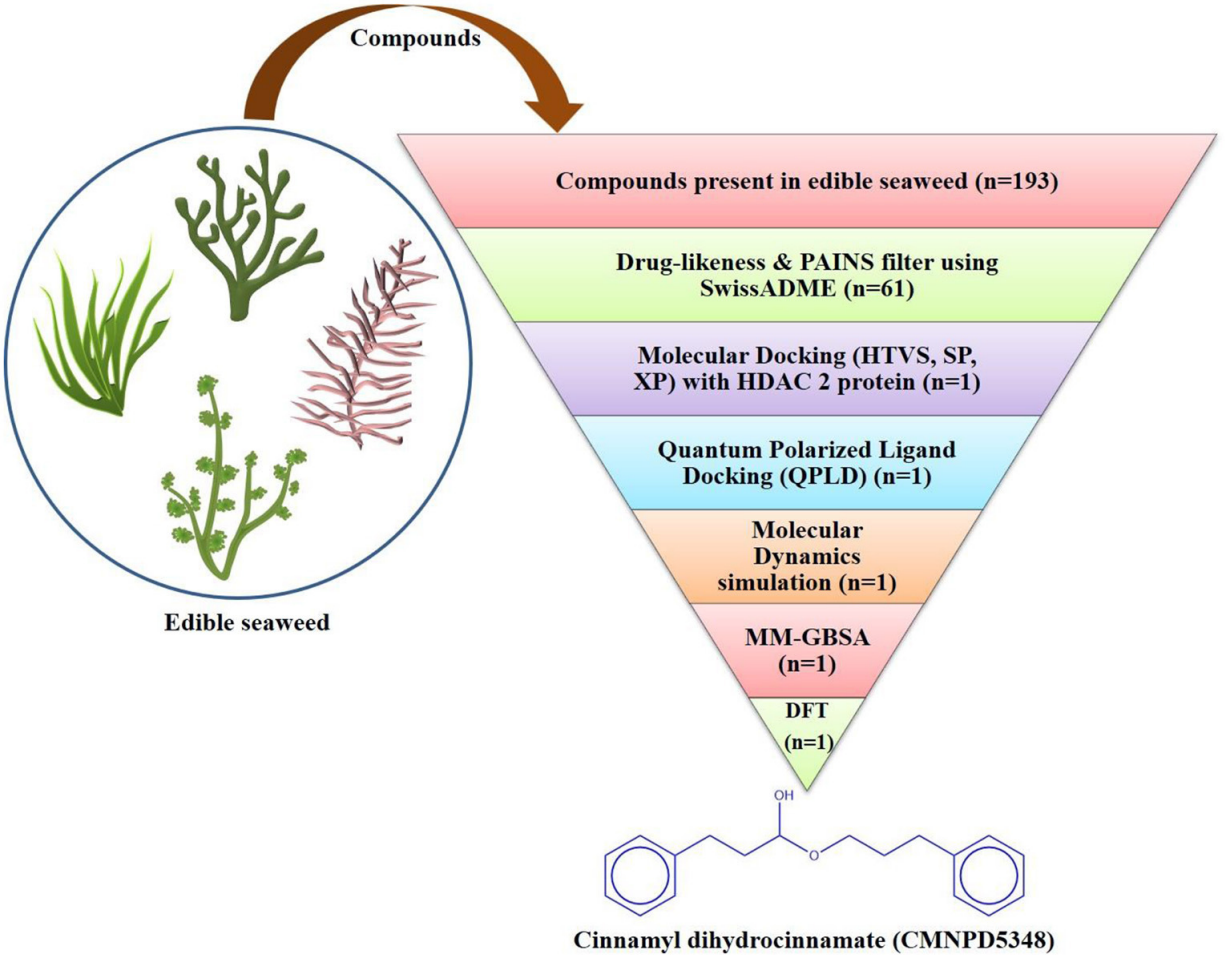
Flowchart of the virtual screening workflow for identification of prospective HDAC 2 inhibitor from edible seaweed.

The toxicity profile of the selected bioactive compound, cinnamyl dihydrocinnamate, from edible seaweed waste was predicted using pkCSM online webserver (http://biosig.unimelb.edu.au/pkcsm/) (64). The selected compound structure was drawn using the Chemdraw drawing tool and was converted into SMILES format for the prediction studies. The possible predicted toxicity risks evaluated were AMES toxicity, hepatotoxicity, skin irritation, max. tolerated dose (human), oral rat acute toxicity (LD50), oral rat chronic toxicity (LOAEL), and hERG I, II inhibitors (Figure 1).

## Result and Discussion

### Drug Likeness Virtual Screening Analysis

The edible seaweed compounds (*n* = 193) extracted from the CMPND database were predicted to evaluate the pharmacokinetics and drug-likeness using the Swiss ADME online webserver (65). The Swiss ADME results give a detailed and extensive physicochemical profile of the edible seaweed compounds. We have filtered all the edible seaweed compounds (*n* = 194) by applying Lipinski's rule of five, Ghosh filter, Veber filter, and PAINS filter (Supplementary File S1). From the total edible seaweed compounds (*n* = 61), molecules are passed (Supplementary File S2) through the filter without any violations. These compounds showed drug-likeness properties. Christopher A. Lipinski created the Lipinski's rule of five (Ro5), a rule of thumb for determining drug-likeness or to determine if a compound with specific pharmacological activity has chemical and physical properties that would make it a likely orally active drug in humans (34, 66). Pan assay interference compounds (PAINS) are physicochemical filters that can predict the pharmacokinetic features of drugs. The PAINS filter assists in determining if a compound is a biological assay response or not (37). Afterward, to screen the HDAC 2 protein inhibitor ligands, we have performed HTVS molecular docking by using the Schrödinger Glide program.

### Glide SP, XP, and QPLD Study

**Figure 2:**
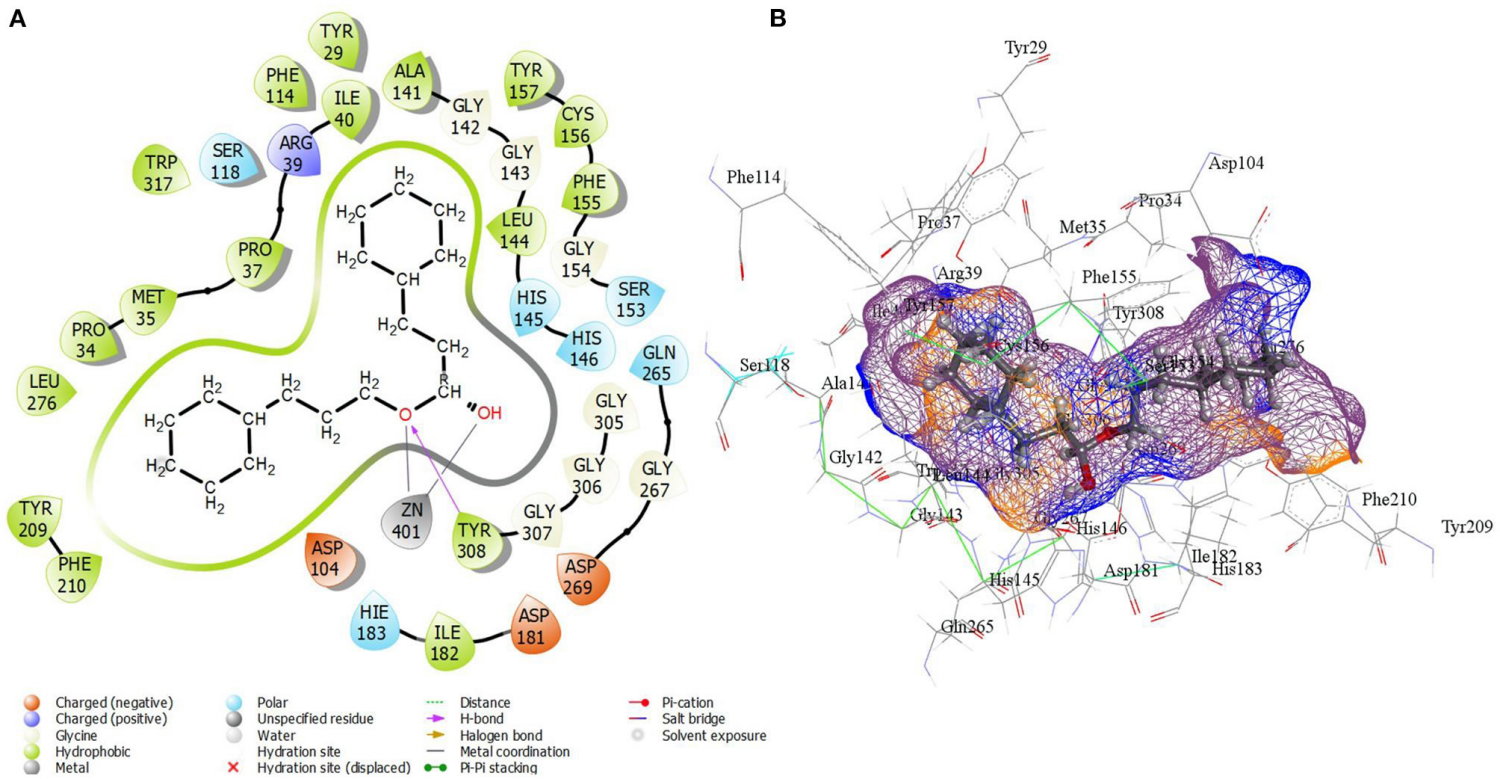
**(A)** 2D molecular interactions and **(B)** 3D interactions in the active site of best-docked compound, cinnamyl dihydrocinnamate (CMNPD5348) with HDAC 2 protein after QPLD docking.

The result of virtual screening revealed the identification of one compound from the edible seaweed as a potential inhibitor as it exhibited non-covalent interactions with the amino acid residues from the catalytic residue of the HDAC 2 protein. The edible seaweed compound dataset containing 61 compounds was initially screened using SwissADME. The high throughput virtual screening module then filtered the 61 edible seaweed compounds. The lower energy score was utilized to rank the top compounds from the dataset. The lower the binding energy, the higher the binding efficiency and, as a result, the stronger the inhibition. The glide scores of seven compounds showing high binding affinity are given in Supplementary Table S3. The XP Gscore was found to be higher in cinnamyl dihydrocinnamate (CMNPD5348) with an energy of −11.829 kcal/mol. Finally, the top hit compound, cinnamyl dihydrocinnamate, is again docked into the binding cavity of HDAC 2 receptor using QPLD. The top hit compound was then subjected to re-dock with HDAC 2 using QPLD for evaluating relative binding interactions and the strength of binding by accurate charge calculation through hybrid quantum mechanics and molecular mechanics method (QM/MM), respectively. The selected compound showed the highest binding affinity toward HDAC 2 with a QPLD DG score of −11.190 kcal/mol (Figures 2A,B). The compound also interacts with the cofactor Zn^2+^ of HDAC 2 protein. Overall, the selected hit compound, cinnamyl dihydrocinnamate's, position midway between the active site channel and the cavity, as well as its direct binding with the catalytic Zn^2+^ ion is optimal for inhibiting HDAC 2 protein, which will prevent deacetylation by interacting with the acetylated lysine residues as a substrate (67). The amino acid residues of the binding cavity within 4 Å that interacts with the selected hit compound were Pro 34, Met 35, Pro 37, Arg 39, Ile 40, Asp 104, Ala 141, Gly 142, Gly 143, Leu 144, His 145, His 146, Tyr 209, Phe 210, Leu 276, Gly 305, Gly 306, Gly 307, Tyr 308, and Asp 401. The hydrophobic zones in the neighborhood of the active site of HDAC 2 is composed of amino acid residues, namely Pro 34, Met 35, Pro 37, Ile 40, Asp 104, Ala 141, Leu 144, Tyr 209, Phe 210, Leu 276, and Tyr 308. Additionally, there were positively charged amino acid residues, namely Arg 39, His 145, and His 146. Binding mode analysis revealed that the compound forms one hydrogen bond interaction with the amino acid residue Tyr 308.

### Molecular Dynamics (MD) Simulation Study

**Figure 3:**
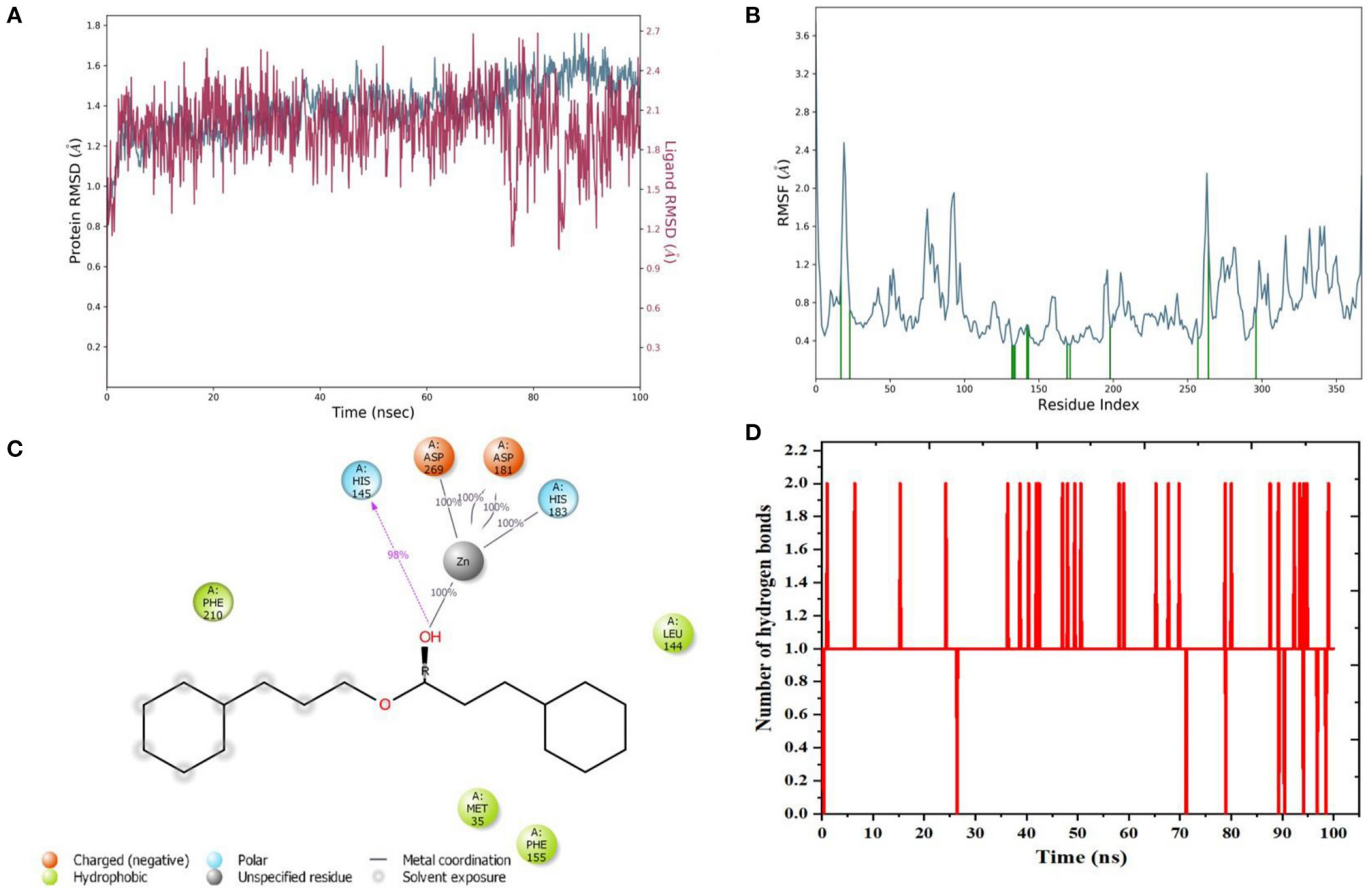
MD simulation analysis of cinnamyl dihydrocinnamate (CMNPD5348) in complex with HDAC 2 during 100 ns MD simulation time **(A)** Plot of root mean square deviations (RMSD) (protein RMSD (C-α atom of HDAC 2 protein) is shown in gray while RMSD of cinnamyl dihydrocinnamate are shown in red) **(B)** plot of root mean square fluctuations (RMSF) values **(C)** plot of ligand interaction in the binding cavity and **(D)** plot of the number of hydrogen bonding interactions.

**Figure 4:**
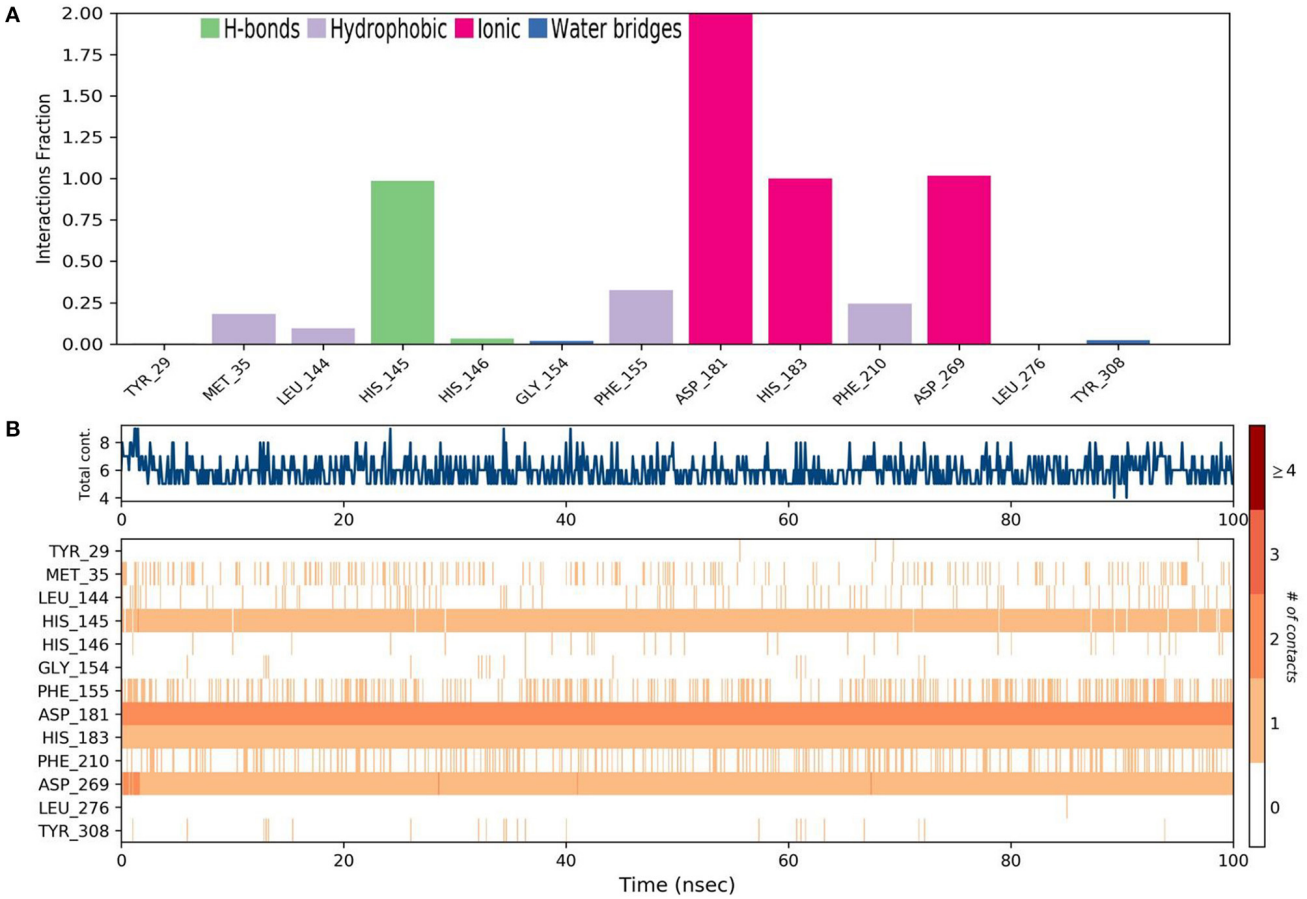
MD simulation analysis of cinnamyl dihydrocinnamate (CMNPD5348) in a complex with HDAC 2 during 100 ns MD simulation time **(A)** stacked bar chart plot of protein–ligand contact analysis. Abscissa represents the amino acid number and ordinate represents interactions fraction **(B)** analysis of total contacts timeline analysis of MD trajectory. Darker shades correspond to a higher number of contacts.

**Figure 5:**
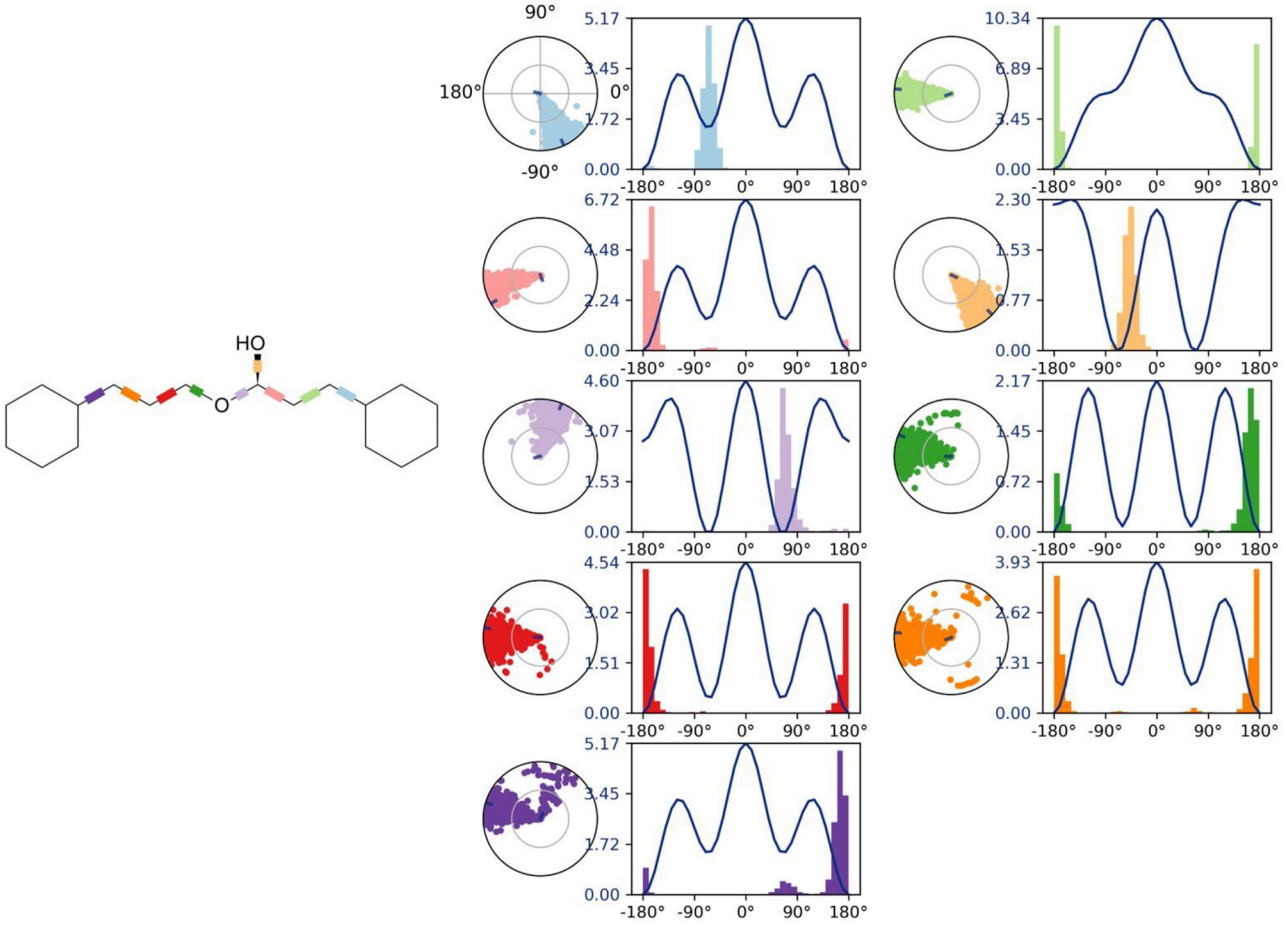
Analysis of the torsional degree of freedom for the rotatable bonds present in cinnamyl dihydrocinnamate (CMNPD5348) during 100 ns of MD simulation.

Molecular dynamics (MD) simulation studies were carried out to determine the stability of cinnamyl dihydrocinnamate bound HDAC 2 protein complex. A simulation of 100 ns displayed stable conformation while studying the root mean square deviation (RMSD) values. The Cα atoms of HDAC 2 protein were used to analyze the RMSD, which was then plotted against the simulation time, as presented in Figure 3A. The cinnamyl dihydrocinnamate when bound to HDAC 2 showed RMSD (Cα atom of HDAC 2) values between 0.7 and 1.7 Å with an average value of 1.39 Å. In the case of ligand, following the initial equilibration fluctuation, the RMSD for cinnamyl dihydrocinnamate stayed in the range of 1–2.6 Å till the end of the simulation. The overall RMSD of the lead compound cinnamyl dihydrocinnamate is within the acceptable range during the 100-ns simulation, confirming the protein–ligand complex stability. Each amino acid residue plays a critical role in the stability of the protein–ligand complex in the MD simulation study. The RMSF is used to evaluate the flexibility of each amino acid residue and how much it moves or changes throughout a simulation period. The RMSF was employed to investigate the fluctuation of the protein–ligand docking complex in the function of time. The RMSF value was assessed from the MD simulation trajectory and is shown in Figure 3B. If the atoms in the active site and the main chain fluctuated minimally, it indicated that the conformational change was minimal, implying that the reported lead compound was firmly bound within the cavity of the target protein binding pocket (61). The RMSF results revealed that the Cα atoms of HDAC 2 protein bound to cinnamyl dihydrocinnamate have a mean RMSF value of 0.077 Å, indicating fewer fluctuations in the complex structure. The RMSF plot of the cinnamyl dihydrocinnamate-HDAC 2 complex revealed that the lead compound cinnamyl dihydrocinnamate made contact with 13 amino acids of the HDAC 2 protein, namely, Tyr 29 (1.1 Å), Met 35 (0.721 Å), Leu 144 (0.355 Å), His 145 (0.34 Å), His 146 (0.353 Å), Gly 154 (0.567 Å), Phe 155 (0.555 Å), Asp 181 (0.361 Å), His 183 (0.341 Å), Phe 210 (0.545 Å), Asp 269 (0.427 Å), Leu 276 (1.487 Å), and Tyr 308 (0.674 Å). The RMSF values of most residues are <2 Å with the exception of the loop regions, C and N terminal, showing that the residue conformation is relatively stable during the simulation. The RMSF plot above undoubtedly shows that the HDAC 2 protein residues bound to cinnamyl dihydrocinnamate remained stable throughout the simulation. From the results, it can be observed that cinnamyl dihydrocinnamate shows the least fluctuation throughout the 100-ns simulation time. From the 2d ligand-interaction diagram, it was observed that the hydroxyl group of the lead compound cinnamyl dihydrocinnamate interacted with three residues of the binding cavity of HDAC 2 protein via Zn^2+^ interaction with the amino acid residues: Asp 181 (100%), His 183 (100%), and Asp 269 (100%; Figure 3C). The amino acid residue His 145 (98%) also interacted with the hydroxyl group of the lead compound (Figure 3C). The binding characteristics of HDAC 2 with the ligand cinnamyl dihydrocinnamate were analyzed by studying the hydrogen bond interactions. The number of intermolecular hydrogen bonds in MD simulation trajectories of cinnamyl dihydrocinnamate bound HDAC 2 complex is shown in Figure 3D. The HDAC 2 complex with cinnamyl dihydrocinnamate forms an average of one hydrogen bond during most of the simulation time. This result confirmed the strong inhibition of HDAC 2 by the compound cinnamyl dihydrocinnamate during MD simulation. The study showed similar interpretations with the molecular docking results of one hydrogen bond within the active site residues in HDAC 2 protein. Hydrogen bonding is significant in drug design research since it influences drug metabolism, adsorption, and specificity (68). In addition, hydrophobic interactions, ionic or polar interactions, and water bridge-hydrogen bonded protein–ligand interactions mediated by a water molecule contribute to the docked complex's stability during simulation. As a result, the intermolecular contact generated between the HDAC 2 protein and the docked compound was retrieved from the respective MD trajectories using the Desmond module's default parameters. Figure 4A demonstrates the protein–ligand contacts. The dominant contribution to the interaction is given by the Asp 181, His 183, and Asp 269 amino acid residue, where ionic interactions form a 2.0 and 1.0 interaction fraction. The other interactions are given by other residues that help the protein–ligand interaction namely, Met 35, Leu 144, His 145, His 146, Gly 154, Phe 155, and Phe 210, and Tyr 308, where the combination of H-bonds, hydrophobic, and water bridges with interaction fractions are ~0.1–1.0. The protein–ligand's total contacts timeline diagram is prepared to study the intermolecular interactions between HDAC 2 residues and the cinnamyl dihydrocinnamate compound (Figure 4B) after performing 100 ns of MD simulation. The top panel displayed a maximum of 8 specific contacts formed between the protein and the ligand throughout simulations. The bottom panel shows that the residues Asp 269, Phe 210, His 183, Asp 181, Phe 155, His 145, Leu 144, and Met 35 showed crucial interactions with the ligand cinnamyl dihydrocinnamate, which is represented by a darker shade. The amino acid residue Met 35, Leu 145, and Phe 210 also showed interaction during our previous molecular docking findings, within the active site residues of the HDAC 2 pocket. To better comprehend the conformation strains, the torsional dynamics for the rotatable bonds present in the lead compound, cinnamyl dihydrocinnamate were studied and depicted in Figure 5. The plot depicted the lead compound simulation trajectory from 0 to 100 ns, as well as the conformational evolution of rotatable bonds (RB). The bonds were color-coded and rotatable bond torsions were illustrated in a two-dimensional (2D) plot. Throughout the simulation, dial charts indicated the torsion's conformation. The time evolution of the simulation is plotted radially outward, with the initiation of the simulation at the center of the radial map. The torsions' probability density is depicted in bar plots, which depict the information received from the dial plots. The kcal/mol numbers on the left *Y*-axis of the graphs are in kcal/mol. Cinnamyl dihydrocinnamate has nine rotatable bonds in total, and the dial plots demonstrated that the rotatable bonds are rotated between 180° and 180° (Figure 5). This demonstrated that the ligand cinnamyl dihydrocinnamate had greater flexibility in binding and maintaining a stable conformation in the HDAC 2 protein active site residue.

### Free Energy Landscape Analysis

**Figure 6:**
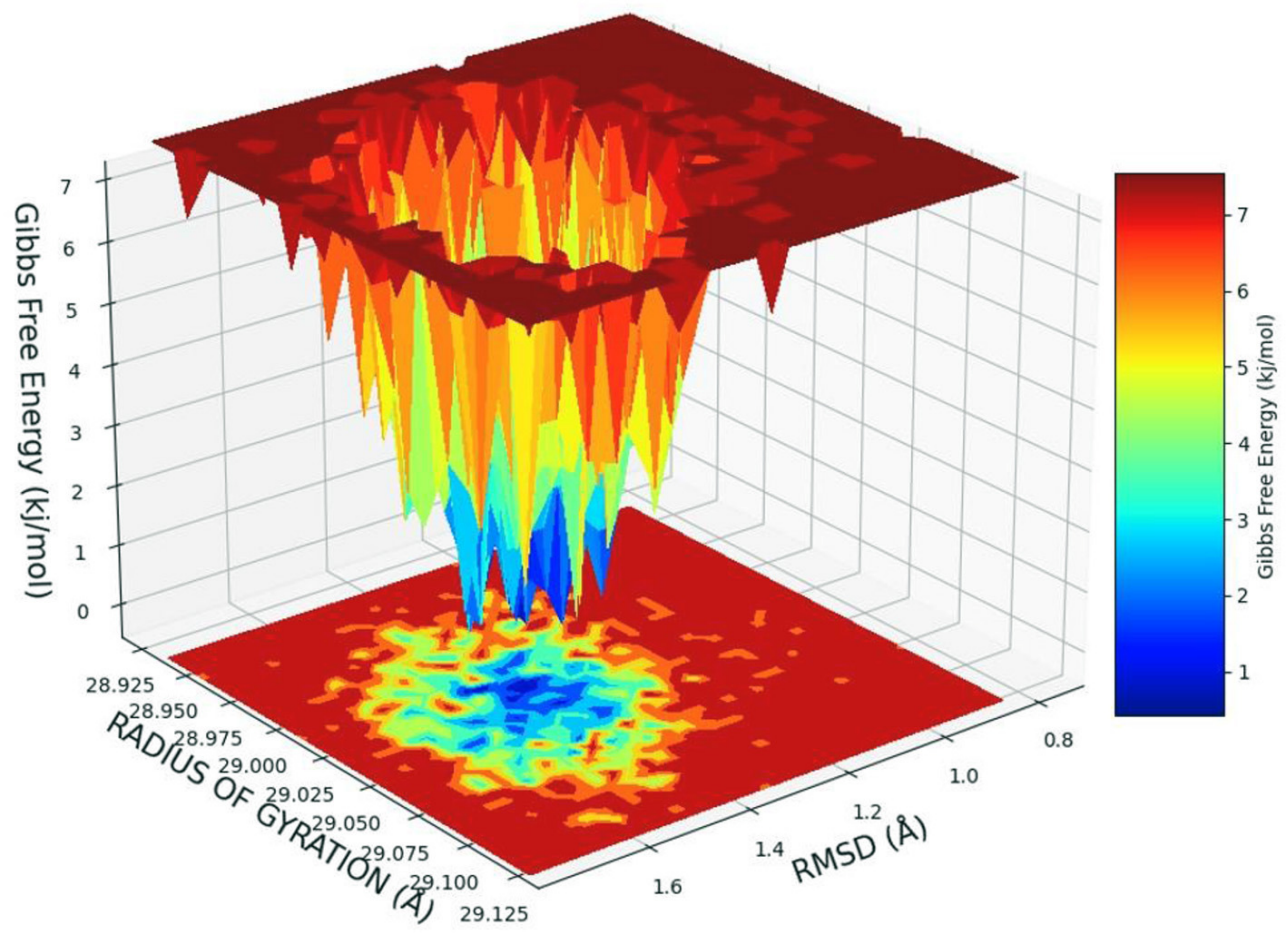
Free energy landscape displaying the achievement of global minima (ΔG, kJ/mol) of HDAC 2 in the presence of cinnamyl dihydrocinnamate (CMNPD5348) concerning their RMSD (Å) and radius of gyration (Rg, Å).

The free energy landscape of (FEL) of achieving global minima of Cα backbone atoms of protein concerning RMSD and radius of gyration (Rg) is displayed in Figure 6. Cinnamyl dihydrocinnamate bound to receptor HDAC 2 achieved the global minima (lowest free energy state) at 1.5 Å RMSD and Rg 29 Å (Figure 6). Due to its great stability and optimal conformation in the bound state of cinnamyl dihydrocinnamate, the FEL was designed for understanding the deterministic behavior of the receptor to the lowest energy state possible. Therefore, FEL is the indicator of protein folding to attain minimum energy state, and that is aptly achieved due to the bound state of ligand cinnamyl dihydrocinnamate. Further free energy landscape (FEL) of cinnamyl dihydrocinnamate-bound receptor complexes exhibited a deep basin over areas of increased free energy with the deep blue color locations (Figure 6) representing the local energy minima and actively promoting the stable conformations, similarly suggested by Singh et al. (69).

### PCA and DCCM of the MD Simulation Trajectories

**Figure 7:**
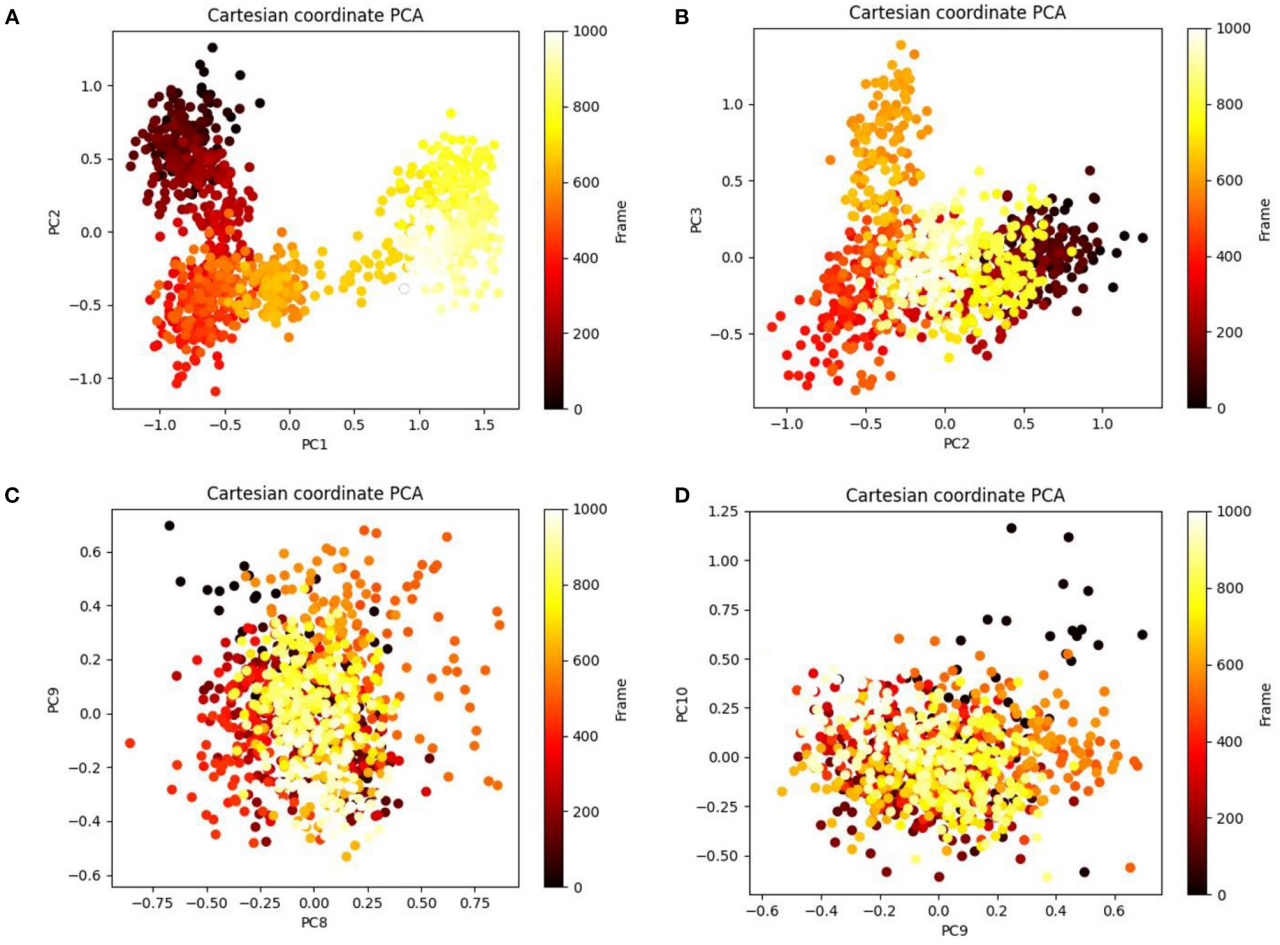
Principal component analysis (PCA) of cinnamyl dihydrocinnamate (CMNPD5348) in complex with HDAC 2 displaying **(A)** PC1 and PC2, **(B)** PC2 and PC3, **(C)** PC8 and PC9, and **(D)** PC9 and PC10 for 100 ns simulation trajectories.

**Figure 8:**
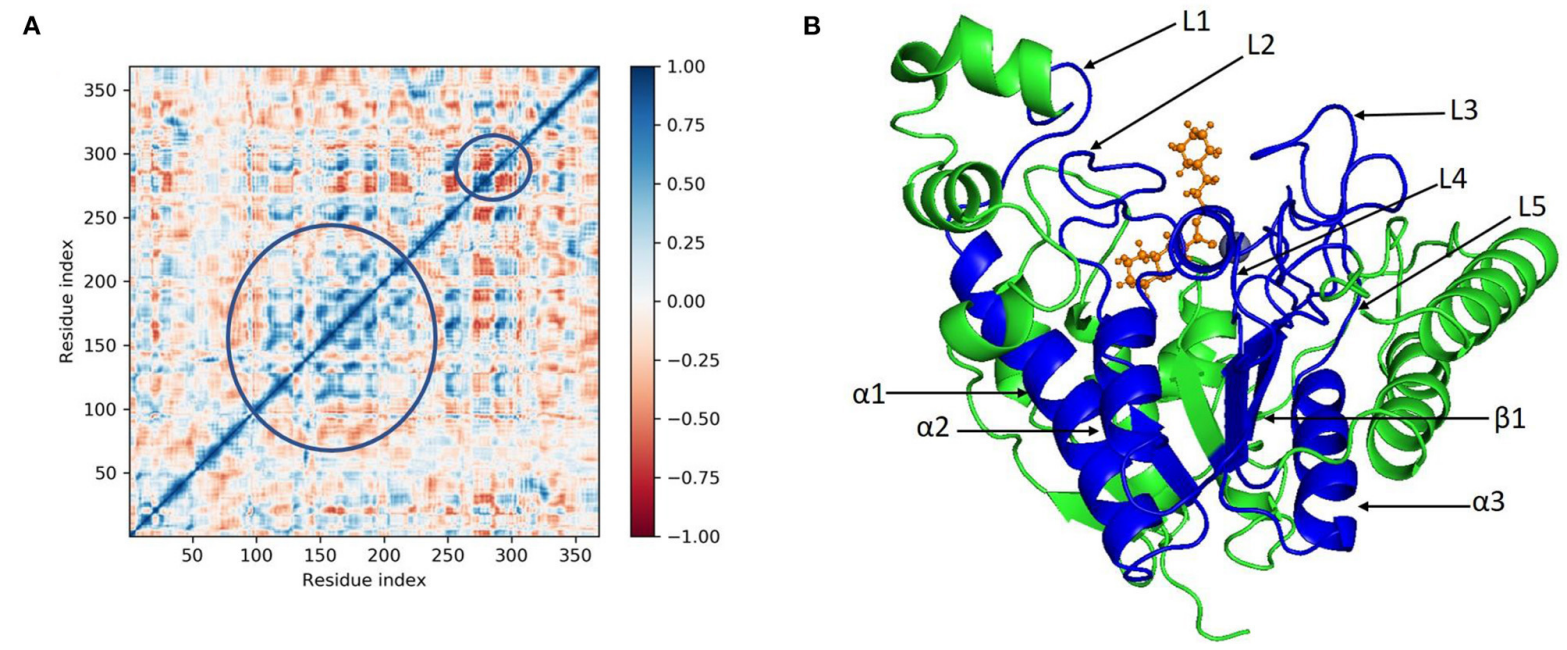
**(A)** Dynamic cross correlation matrix (DCCM) and **(B)** correlated amino acids conformed into secondary structural domains (colored; blue) and non-correlated domains (green) of cinnamyl dihydrocinnamate (CMNPD5348) in complex with HDAC 2 from MD simulation trajectories.

Principal component analysis of the MD simulation trajectories for cinnamyl dihydrocinnamate bound to HDAC 2 was analyzed to interpret the randomized global motion of the atoms of amino acid residues. This analysis interprets the more flexible scattered trajectories owing to the distortion of the protein structure. The internal coordinates' mobility into three-dimensional space in the spatial time of 100 ns was recorded in a covariance matrix, and rational motion of each trajectory is interpreted in the form of orthogonal sets or eigenvectors. The Cα atoms of HDAC 2 from molecular dynamics simulation bound to cinnamyl dihydrocinnamate displayed unordered clustering in PC1 and PC2 modes (Figure 7A). Following this PC2 and PC3 modes displayed better order eigenvalues for the trajectories (Figure 7B). The eigenvectors displayed a less scattered but more positive correlation cluster of the last 50 trajectories (Figure 7C). Most of the trajectories finally settled in PC9 and PC10 (Figure 7D), where the global motion was centered toward the origin of the plot. The global movement of the MD trajectories toward positive correlation eigenvector indicated the compact and converged structure of HDAC 2 complex with cinnamyl dihydrocinnamate. The high periodic global motion was observed along the PC9 and PC10 planes (Figure 7D) due to the grouping of trajectories in a single cluster at the center of the PCA plot. Centering of the trajectories in a single cluster indicates the periodic motion of MD trajectories due to stable conformational global motion. Ordered orientation signifies that the ordered global motion of the trajectories arose due to a stable converged structure. DCCM was generated to analyze the correlative motion of structural domains to attain a stable conformation of the compound after HDAC 2 binding from MD trajectories (Figures 8A,B). The matrix plot's different color codings represent different levels of correlation. The blue color represents a high level of positive correlation, while the red color suggests less or negative correlation among structural domains. The correlation scores on the central mean line (blue) displayed two distinct blocks in cinnamyl dihydrocinnamate (Figure 8A) bound HDAC 2, having a high correlation of movement of the amino acids in HDAC 2 (Figure 8B). In HDAC 2, bounded with the ligand cinnamyl dihydrocinnamate, α helix, β pleated sheet, and loop amino acid residues (100–240) displayed high positive correlation (blue) in three α-helix (α1, α2, α3), one β1 sheet (β1), and five loops (L1, L2, L3, L4, and L5) (Figure 8B). Thus, the DCCM analysis confirmed the previously obtained RMSF result. By studying the dynamics cross-correlation matrix, a similar type of work was earlier reported (59).

### MM-GBSA Calculations

To assess the binding free energy of ligands with HDAC 2 protein molecules, the MM-GBSA technique is commonly used (30, 58). The binding free energy of HDAC 2–cinnamyl dihydrocinnamate complex, as well as the impact of other non-bonded interactions energies were calculated (Table 2). With HDAC 2, the ligand cinnamyl dihydrocinnamate has a binding energy of −11.46 ± 4.91 kcal/mol. The non-bonded interactions like ΔG_bind_Lipo, ΔG_bind_Covalent, ΔG_bind_Hbond, ΔG_bind_Coulomb, ΔG_bind_SolvGB, and ΔG_bind_vdW govern the binding free energy of ΔG_bind_. Across all types of interactions, ΔG_bind_vdW, ΔG_bind_Lipo, ΔG_bind_Coulomb, and ΔG_bind_Hbond contributed the most to the average binding energy (Table 2) in the protein–ligand interaction. But ΔG_bind_SolvGB and ΔG_bind_Covalent have exhibited unfavorable energy contributions and so opposed binding. Thus, the MM-GBSA calculations well-validated the binding energy estimates obtained from molecular docking.

**Table 2:** Binding free energy components for the docking complexes of HDAC 2 protein with ligand cinnamyl dihydrocinnamate (CMNPD5348) calculated by MM-GBSA analysis.

**MM-GBSA (kcal/mol)**	**HDAC 2**
	**Binding free energy values (kcal/mol)**
ΔG_bind_	−11.46 ± 4.91
ΔG_bind_Lipo	−22.73 ± 1.74
ΔG_bind_vdW	−46.12 ± 1.59
ΔG_bind_Coulomb	−13.55 ± 1.44
ΔG_bind_H_bond_	−0.57 ± 0.06
ΔG_bind_SolvGB	70.43 ± 4.30
ΔG_bind_Covalent	1.09 ± 0.26

*Results are calculated in mean ± SD*.

### ADME Property Analysis

The QikProp tool was used to evaluate the ADME properties of the compound cinnamyl dihydrocinnamate in this study. In this work, the physicochemical properties and biological roles are discussed. The ADME analysis is a crucial method for determining the efficacy of a potential pharmaceutical compound. The physicochemical and biological properties investigated included molecular weight, # Stars, SASA, FOSA, FISA, PISA, WPSA, Dipole, Donor H-bond, Acceptor H-bond, QPlogPo/w, QPlogS, QPlogkhsa, QplogBB, QPPCaco, percent human oral absorption, rule of five, and rule of three effects. The values for these descriptors are shown in Table 3. The expected ADME values for the compound cinnamyl dihydrocinnamate are all within the approved range, indicating better drug likeliness and pharmacokinetic characteristics.

**Table 3:** ADME prediction of cinnamyl dihydrocinnamate (CMNPD5348).

**Properties and functions**	**Predictive results**	**Recommended range**
Molecular weight (Da)	266.339	130–725
#Stars	4	0–5
SASA (total solvent accessible surface area)	290.355	300–1,000
FOSA (Hydrophobic component of the SASA)	281.229	0.0–750.0
FISA (Hydrophilic component of the SASA)	9.127	7.0–330.0
PISA (π component of the SASA)	0	0.0–450.0
WPSA (Weakly polar component of the SASA)	0	0.0–175.0
Dipole	0	1.0–12.5
Donor H-bond	0	0–6.0
Acceptor H-bond	4	2.0–20.0
QPlogPo/w (predicted octanol/water coefficient)	0.742	−2–6.5
QPlogS (predicted aqueous solubility)	0.375	−6.5–0.5
QPlogkhsa (binding prediction to human serum albumin)	−0.913	−3–1.2
QplogBB (predicted blood brain/blood partition coefficient)	0.512	−3.0–1.2
QPPCaco (Predicted apparent Caco-2 cell permeability in nm/sec)	8116.211	<25 poor, >500 great
Rule of five	0	Max 4
Rule of three	0	Max 3
% Human oral absorption	100	>80% is high <25% is poor

### Toxicity Prediction of Cinnamyl Dihydrocinnamate

The predicted values related to the AMES toxicity and hepatotoxicity revealed that the cinnamyl dihydrocinnamate was nontoxic and non-mutagenic (Table 3). The liver is associated with energy exchanges as well as xenobiotic and drug biotransformation. Damage to the liver leads to disruption in normal metabolism and can possibly lead to liver failure (70). The hepatotoxicity description predicted that the compounds would not be toxic to the liver. Inhibition of potassium channels encoded by hERG-I and II resulted in fatal ventricular arrhythmia. Inhibition of hERG-I and II resulted in the withdrawal of many compounds from the pharmaceutical industry (71). The selected compound has shown no inhibition of hERG-I and II. However, the selected compound predicted skin sensitization. The compound may induce an allergic reaction when applied dermally. The toxicity analysis server predicted LD50, the lowest-observed-adverse-effect level (LOAEL) and the maximum tolerated dosage range of the selected compound, and the predicted scores are presented in Table 4. It is worth noting that the oral toxicity of the compound evaluated by this tool corresponds to the toxicity risk observed in animal models. The maximum recommended human tolerated dose (MRTD) (log mg/kg/day) bestowed the toxic dose threshold of chemicals in humans in phase I clinical trials. It is considered low when ≤ 0.477 log mg/kg/day and high >0.477 log mg/kg/day. Our findings showed that the compound has high MRTD.

**Table 4:** *In-silico* toxicity predicted values of cinnamyl dihydrocinnamate using pkCSM.

**Sl** **No**.	**Predicted parameters**	**Predicted** **value**	**Unit**
1.	AMES toxicity	No	Categorical (Yes/No)
2.	Max. tolerated dose (human)	0.924	Numeric (log mg/kg/day)
3.	hERG I inhibitor	No	Categorical (Yes/No)
4.	hERG II inhibitor	No	Categorical (Yes/No)
5.	Oral rat acute toxicity (LD50)	1.807	Numeric (mol/kg)
6.	Oral rat chronic toxicity (LOAEL)	2.272	Numeric (log mg/kg_bw/day)
7.	Hepatotoxicity	No	Categorical (Yes/No)
8.	Skin sensitisation	Yes	Categorical (Yes/No)

**Figure 9:**
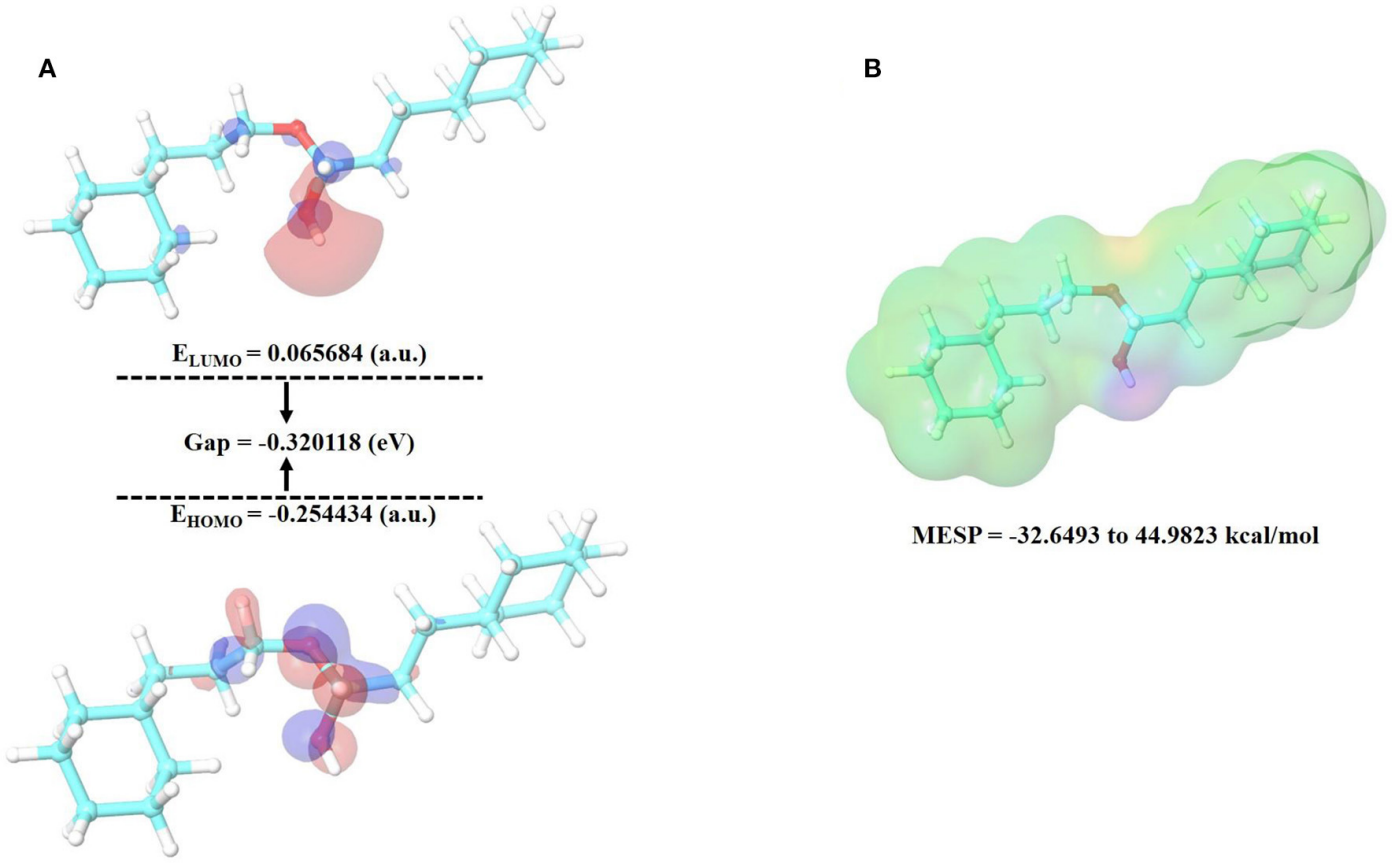
**(A)** HOMO, LUMO and **(B)** MESP of cinnamyl dihydrocinnamate (CMNPD5348).

Density functional theory. The electronic structures of compounds are linked to their pharmacological activities. The energy and distribution patterns of their orbitals, specifically the highest occupied molecular orbital (HOMO) and the lowest unoccupied molecular orbital (LUMO), which are commonly referred to as frontier molecular orbitals (FMOs), are also crucial to explore (72). Frontier molecular orbitals were used to analyze the compounds' electrical characteristics and their electron transport potential. The biological activity and molecular characteristics of the substances are determined by these orbitals. To reveal insight on the electronic structural properties of our selected compound, cinnamyl dihydrocinnamate, we have optimized the compound structure utilizing the density functional theory (DFT) calculation using B3LYP/6-31G^**^ basic set (single point energy calculation). The HOMO is the highest-energy (electron-rich) orbital that has the potential to provide electrons, whereas the LUMO is the lowest-lying empty orbital (lack of electron) that has the capacity to accept electrons (73). Figures 9A,B illustrate the energy of HOMO and LUMO, their energy gap (E), and the molecular electrostatic potential surface (MESP) of the studied compound. The LUMO and HOMO energies influence how a compound interacts with other species, as well as its chemical reactivity and kinetic stability. Low-band energy gaps (ΔE), which signify stronger compound reactivity with the receptor, were indicated by the smallest difference between LUMO and HOMO. Since the LUMO and HOMO are responsible for the charges exchanged in a chemical reaction, a lower band energy gap (ΔE) indicates more reactivity of the compound. The decrease in energy gap values corresponds to a rise in the order of reactivity (60, 74–76). A Lower energy gap was observed with the selected compound, cinnamyl dihydrocinnamate (CMNPD5348) (ΔE = – 0.320118 eV) (Figure 9A). In cinnamyl dihydrocinnamate, HOMO localizes on electronegative atoms, and highly delocalized HOMO suggests that electrons can move around the molecule more freely, resulting in better intramolecular charge transfer. The presence of negative HOMO–LUMO values in our chosen compound indicates high stability, which is required to establish a stable protein–ligand interaction. More energy gap led to a weak affinity of the protein–ligand interaction. Furthermore, the energy gap is a good method for identifying the most active molecules. The intermolecular charge transfer and bioactivity of a compound are significantly influenced by the energy gap between the HOMO and LUMO energies (61, 74). The molecular electrostatic potential (MEP) plot can be used to gain insight into a compound's three-dimensional structural and topological properties. It identifies the reactive sites for electrophilic and nucleophilic attack in a compound for binding to protein in protein–ligand interaction, as well as the charge distribution (positive and negative) in the selected compound (72). The various charges are represented by different colors. The red color denotes negative potential (negative regions), which has a propensity for attracting protons; the blue color denotes positive potential (positive regions), which has an attraction for repelling protons; and the green color denotes zero potential. Potential increases were denoted as red<orange<yellow<green<blue. To make a hydrogen bond with the protein, the negative region is crucial (60, 74, 77). In our selected compound, the blue color (positive region) is situated around all surfaces of the compound, which indicates their potentiality to act as a H-bond donor in protein–ligand interactions, whereas highly negative regions were situated at the oxygen atom. This region may be responsible for the protein's hydrogen-bonding interactions. These results corroborate our molecular docking finding. In our selected compound, the blue color (positive region) indicates their potentiality to act as the H-bond donor in protein–ligand interactions. These results corroborate our molecular docking finding.

## Conclusions

Overexpression of HDAC 2, a class I HDAC isoform, has been discovered in a range of cancers. In a growing number of research articles, data, and proof, the role of HDAC 2 in cancer development and progression has been established. The primary goal of designing HDAC specific inhibitors is to avoid non-selective interactions with other HDAC classes, which can result in substantial side effects. In this study, we have employed a computational-based drug design workflow to recognize potent HDAC 2 inhibitors. To investigate potential HDAC 2 inhibitors as lead compounds from edible seaweed compounds, we used a combination of *in silico* methodologies such as drug-likeness, molecular docking, molecular dynamics simulation, MM-GBSA, PCA, DCCM, free energy landscape, and density functional theory based virtual screening. The lead compound cinnamyl dihydrocinnamate (CMNPD5348) interacted with HDAC 2 protein efficiently according to our findings. Molecular dynamics simulations and binding free energy calculations revealed that the docking complex has a high level of binding stability and a low binding free energy. At 100 ns of molecular dynamics simulation time, the metal ion Zn and ligand interaction was also observed, which is crucial for inhibiting the HDAC 2 protein's enzymatic activity. The hit compound also showed high pharmacokinetic characteristics in the drug-likeness and ADME analysis, indicating that it could be a promising lead candidate. Cinnamyl dihydrocinnamate has been identified in edible seaweed *Caulerpa racemosa*, which is mainly found in shallow water locations around the world. In this study, the compounds were discovered to be effective HDAC 2 inhibitors. This discovery is expected to lead to the development of efficient HDAC 2 inhibitors for cancer therapy. The fishing industry generates a lot of seaweed debris, as well as other substances. This study will motivate researchers to look into and extract beneficial compounds from edible seaweed waste for biomedical research and therapeutic applications. This study will also provide an important step in the exploration of bioactive compounds from seaweed waste. In addition, the outcomes of *in-vitro* and *in-vivo* analysis will be necessary to validate the molecular docking, molecular dynamics simulation, and DFT investigations.

## Data Availability Statement

The original contributions presented in the study are included in the article/Supplementary Materials, further inquiries can be directed to the corresponding authors.

## Author Contributions

KB, IA, SP, BR, TS, and DB: conceived and designed the experiments. KB, IA, SP, BR, TS, HP, AG, DB, and MA: writing—original draft preparation. KB, IA, SP, HE, WW, and MA: formatting and editing according journal guidelines. KB, IA, BR, TS, HP, AG, DB, ZA, HE, and WW: writing—review and editing. All authors have read and agreed to the published version of the manuscript.

## Conflict of Interest

SP is employed by NatNov Bioscience Private Limited. The remaining authors declare that the research was conducted in the absence of any commercial or financial relationships that could be construed as a potential conflict of interest.

## Publisher's Note

All claims expressed in this article are solely those of the authors and do not necessarily represent those of their affiliated organizations, or those of the publisher, the editors and the reviewers. Any product that may be evaluated in this article, or claim that may be made by its manufacturer, is not guaranteed or endorsed by the publisher.

## References

[1] Pati S Chatterji A Dash BP Nelson BR Sarkar T Shahimi S. Structural characterization and antioxidant potential of chitosan by γ-irradiation from the carapace of horseshoe crab. Polymers. (2020) 12:2361. 10.3390/polym12102361 33076234 PMC7602389

[2] Pati S Sarkar T Sheikh HI Bharadwaj KK Mohapatra PK Chatterji A. γ-irradiated chitosan from carcinoscorpius rotundicauda (Latreille, 1802) improves the shelf life of refrigerated aquatic products. Front Mar Sci. (2021) 8:498. 10.3389/fmars.2021.664961

[3] Šimat V. Nutraceuticals and pharmaceuticals from marine fish and invertebrates. Mar Drugs. (2021) 19:10–2. 10.3390/md19070401 34356826 PMC8304407

[4] Ghosh S Sarkar T Pati S Kari ZA Edinur HA Chakraborty R. Novel bioactive compounds from marine sources as a tool for functional food development. Front Mar Sci. (2022) 9:832957. 10.3389/fmars.2022.832957

[5] Alencar POC Lima GC Barros FCN Costa LEC Ribeiro CVPE Sousa WM. A novel antioxidant sulfated polysaccharide from the algae Gracilaria caudata: *in vitro* and *in vivo* activities. Food Hydrocoll. (2019) 90:28–34. 10.1016/j.foodhyd.2018.12.007

[6] Ghosh S Sarkar T Das A Chakraborty R. Natural colorants from plant pigments and their encapsulation: an emerging window for the food industry. LWT. (2022) 153:112527. 10.1016/j.lwt.2021.112527

[7] Menaa F Wijesinghe U Thiripuranathar G Althobaiti NA Albalawi AE Khan BA. Marine algae-derived bioactive compounds: a new wave of nanodrugs? Mar. Drugs. (2021) 19:1–36. 10.3390/md19090484 34564146 PMC8469996

[8] Li K Li X-M Gloer JB Wang B-G. Isolation, characterization, and antioxidant activity of bromophenols of the marine red alga Rhodomela confervoides. J Agric Food Chem. (2011) 59:9916–21. 10.1021/jf2022447 21838299

[9] Haq SH Al-Ruwaished G Al-Mutlaq MA Naji SA Al-Mogren M Al-Rashed S. Antioxidant, anticancer activity and phytochemical analysis of green algae, chaetomorpha collected from the Arabian Gulf. Sci Rep. (2019) 9:18906. 10.1038/s41598-019-55309-1 31827196 PMC6906397

[10] Liu Z Gao T Yang Y Meng F Zhan F Jiang Q. Anti-cancer activity of porphyran and carrageenan from red seaweeds. Molecules. (2019) 24:4286. 10.3390/molecules24234286 31775255 PMC6930528

[11] Lozano Muñoz I Díaz NF. Minerals in edible seaweed: health benefits and food safety issues. Crit Rev Food Sci Nutr. (2022) 62:1592–607. 10.1080/10408398.2020.1844637 33203217

[12] Moga MA Dima L Balan A Blidaru A Dimienescu OG Podasca C. Are bioactive molecules from seaweeds a novel and challenging option for the prevention of HPV infection and cervical cancer therapy?-A review. Int J Mol Sci. (2021) 22:629. 10.3390/ijms22020629 33435168 PMC7826946

[13] Chinnari S Pati S Dash BP Chatterji A. Seaweeds – promising organic fertilizers. Sci Rept. (2016) 2–4.

[14] Gibilisco PE Lancelotti JL Negrin VL Idaszkin YL. Composting of seaweed waste: evaluation on the growth of *Sarcocornia perennis*. J Environ Manage. (2020) 274:111193. 10.1016/j.jenvman.2020.111193 32810680

[15] Rammou E Mitani A Ntalos G Koutsianitis D Taghiyari HR Papadopoulos AN. The potential use of seaweed (*Posidonia oceanica*) as an alternative lignocellulosic raw material for wood composites manufacture. Coatings. (2021) 11:69. 10.3390/coatings11010069

[16] The global burden of adolescent and young adult cancer in 2019: a systematic analysis for the Global Burden of Disease Study 2019. LancetOncol. 23, (2022) 27–52. 10.1016/S1470-2045(21)00581-7 34871551 PMC8716339

[17] Bharadwaj KK Rabha B Pati S Choudhury BK Sarkar T Gogoi SK. Green synthesis of silver nanoparticles using diospyros malabarica fruit extract and assessments of their antimicrobial, anticancer and catalytic reduction of 4-nitrophenol (4-NP). Nanomater. (2021) 1:1999. 10.3390/nano11081999 34443829 PMC8401075

[18] Bharadwaj KK Rabha B Pati S Sarkar T Choudhury BK Barman A. Green synthesis of gold nanoparticles using plant extracts as beneficial prospect for cancer theranostics. Molecules. (2021) 26:6389. 10.3390/molecules26216389 34770796 PMC8586976

[19] Rabha B Bharadwaj KK Baishya D Sarkar T Edinur HA Pati S. Synthesis and characterization of diosgenin encapsulated poly-ε-caprolactone-pluronic nanoparticles and its effect on brain cancer cells. Polymers. (2021) 13:1322. 10.3390/polym13081322 33919483 PMC8073865

[20] Rabha B Bharadwaj KK Pati S Choudhury BK Sarkar T Kari ZA. Development of polymer-based nanoformulations for glioblastoma brain cancer therapy and diagnosis: an update. Polymers 13:4114. 10.3390/polym13234114 34883617 PMC8659151

[21] Pádua D Rocha E Gargiulo D Ramos AA. Bioactive compounds from brown seaweeds: phloroglucinol, fucoxanthin and fucoidan as promising therapeutic agents against breast cancer. Phytochem Lett. (2015) 14:91–8. 10.1016/j.phytol.2015.09.007

[22] Sun X Zhong Y Luo H Yang Y. Selenium-containing polysaccharide-protein complex in Se-enriched ulva fasciata induces mitochondria-mediated apoptosis in A549 human lung cancer cells. Mar Drugs. (2017) 15:215. 10.3390/md15070215 28714901 PMC5532657

[23] Olivares-Bañuelos T Gutiérrez-Rodríguez AG Méndez-Bellido R Tovar-Miranda R Arroyo-Helguera O Juárez-Portilla C. Brown seaweed egregia menziesii's cytotoxic activity against brain cancer cell lines. Molecules. (2019) 24:260. 10.3390/molecules24020260 30641974 PMC6359252

[24] Lin Y Qi X Liu H Xue K Xu S Tian Z. The anti-cancer effects of fucoidan: a review of both *in vivo* and *in vitro* investigations. Cancer Cell Int. (2020) 20:154. 10.1186/s12935-020-01233-8 32410882 PMC7206694

[25] Ho TCS Chan AHY Ganesan A. Thirty Years of HDAC Inhibitors: 2020 Insight and Hindsight. J Med Chem. (2020) 63:12460–84. 10.1021/acs.jmedchem.0c00830 32608981

[26] Yang H Sun B Xu K He Y Zhang T Hall SRR. Pharmaco-transcriptomic correlation analysis reveals novel responsive signatures to HDAC inhibitors and identifies Dasatinib as a synergistic interactor in small-cell lung cancer. eBioMedicine. (2021) 69:103457. 10.1016/j.ebiom.2021.103457 34224975 PMC8264109

[27] Sixto-López Y Gómez-Vidal JA de Pedro N Bello M Rosales-Hernández MC Correa-Basurto J. Hydroxamic acid derivatives as HDAC1, HDAC6 and HDAC8 inhibitors with antiproliferative activity in cancer cell lines. Sci Rep. (2020) 10:10462. 10.1038/s41598-020-67112-4 32591593 PMC7320180

[28] Shetty MG Pai P Deaver RE Satyamoorthy K Babitha KS. Histone deacetylase 2 selective inhibitors: a versatile therapeutic strategy as next generation drug target in cancer therapy. Pharmacol Res. (2021) 170:105695. 10.1016/j.phrs.2021.105695 34082029

[29] Pettersen EF Goddard TD Huang CC Couch GS Greenblatt DM Meng EC. UCSF Chimera—a visualization system for exploratory research and analysis. J Comput Chem. (2004) 25:1605–12. 10.1002/jcc.20084 15264254

[30] Schrödinger Release 2021-4: Prime. New York, NY: LLC (2021).

[31] Schrödinger Release Maestro 2021-4. Schrödinger, LLC (2021).

[32] Jorgensen WL Tirado-Rives J. The OPLS [optimized potentials for liquid simulations] potential functions for proteins, energy minimizations for crystals of cyclic peptides and crambin. J Am Chem Soc. (1988) 110:1657–66. 10.1021/ja00214a001 27557051

[33] Lipinski CA. Drug-like properties and the causes of poor solubility and poor permeability. J Pharmacol Toxicol Methods. (2000) 44:235–49. 10.1016/S1056-8719(00)00107-6 11274893

[34] Lipinski CA Lombardo F Dominy BW Feeney PJ. Experimental and computational approaches to estimate solubility and permeability in drug discovery and development settings1PII of original article: S0169-409X(96)00423-1. The article was originally published in Advanced Drug Delivery Reviews 23 (1997) 3. Adv Drug Deliv Rev. (2001) 46:3–26. 10.1016/S0169-409X(96)00423-1 11259830

[35] Ghose AK Viswanadhan VN Wendoloski JJ. A Knowledge-based approach in designing combinatorial or medicinal chemistry libraries for drug discovery. 1 A qualitative and quantitative characterization of known drug databases. J Comb Chem. (1999) 1:55–68. 10.1021/cc9800071 10746014

[36] Veber DF Johnson SR Cheng H-Y Smith BR Ward KW Kopple KD. Molecular properties that influence the oral bioavailability of drug candidates. J Med Chem. (2002) 45:2615–23. 10.1021/jm020017n 12036371

[37] Baell JB Holloway GA. New substructure filters for removal of pan assay interference compounds (PAINS) from screening libraries and for their exclusion in bioassays. J Med Chem. (2010) 53:2719–40. 10.1021/jm901137j 20131845

[38] Sarkar T Bharadwaj KK Salauddin M Pati S Chakraborty R. Phytochemical characterization, antioxidant, anti-inflammatory, anti-diabetic properties, molecular docking, pharmacokinetic profiling, and network pharmacology analysis of the major phytoconstituents of raw and differently dried *Mangifera indica* (Himsaga). Appl Biochem Biotechnol. (2021) 194:950–87. 10.1007/s12010-021-03669-8 34591254

[39] Mahmud S Uddin MAR Paul GK Shimu MSS Islam S Rahman E. Virtual screening and molecular dynamics simulation study of plant-derived compounds to identify potential inhibitors of main protease from SARS-CoV-2. Brief Bioinform. (2021) 22:1402–14. 10.1093/bib/bbaa428 33517367 PMC7929365

[40] Khalaf RA Al-Rawashdeh S Sabbah D Abu Sheikha G. Molecular docking and pharmacophore modeling studies of fluorinated benzamides as potential CETP inhibitors. Med Chem. (2017) 13:239–53. 10.2174/1573406412666161104121042 27823564

[41] Tripathi SK Selvaraj C Singh SK Reddy KK. Molecular docking, QPLD, and ADME prediction studies on HIV-1 integrase leads. Med Chem Res. (2012) 21:4239–51. 10.1007/s00044-011-9940-6

[42] Schrödinger Release 2022-1. Schrödinger Release, 2022-1: Desmond Molecular Dynamics System, D. E. Shaw Research, New York, NY, 2021. Maestro-Desmond Interoperability Tools. New York, NY: Schrödinger (2021).

[43] Acar Çevik U Celik I Işik A Ahmad I Patel H Özkay Y. Design, synthesis, molecular modeling, DFT, ADME and biological evaluation studies of some new 1,3,4-oxadiazole linked benzimidazoles as anticancer agents and aromatase inhibitors. J Biomol Struct Dyn. (2022). 10.1080/07391102.2022.2025906. [Epub ahead of print]. 35037830

[44] Ayipo YO Ahmad I Najib YS Sheu SK Patel H Mordi MN. Molecular modelling and structure-activity relationship of a natural derivative of o-hydroxybenzoate as a potent inhibitor of dual NSP3 and NSP12 of SARS-CoV-2: *in silico* study. J Biomol Struct Dyn. (2022). 10.1080/07391102.2022.2026818. [Epub ahead of print]. 35037841

[45] Boulaamane Y Ahmad I Patel H Das N Britel MR Maurady A. Structural exploration of selected C6 and C7-substituted coumarin isomers as selective MAO-B inhibitors. J Biomol Struct Dyn. (2022) 1–15. 10.1080/07391102.2022.2033643 35168478

[46] Mark P Nilsson L. Structure and dynamics of the TIP3P, SPC, and SPC/E water models at 298 K. J Phys Chem A. (2001) 105:9954–60. 10.1021/jp003020w

[47] Jorgensen WL Chandrasekhar J Madura JD Impey RW Klein ML. Comparison of simple potential functions for simulating liquid water. J Chem Phys. (1983) 79:926–35. 10.1063/1.445869

[48] Jorgensen WL Maxwell DS Tirado-Rives J. Development and testing of the OPLS All-atom force field on conformational energetics and properties of organic liquids. J Am Chem Soc. (1996) 118:11225–36. 10.1021/ja9621760

[49] Bowers KJ Chow DE Xu H Dror RO Eastwood MP Gregersen BA. Scalable algorithms for molecular dynamics simulations on commodity clusters. In: SC'06: Proceedings of the 2006 ACM/IEEE Conference on Supercomputing. (2006). p. 43. 10.1145/1188455.1188544

[50] Chow E Rendleman CA Bowers KJ Dror RO Gullingsrud J Sacerdoti FD. Desmond Performance on a Cluster of Multicore Processors (2008).

[51] Shivakumar D Williams J Wu Y Damm W Shelley J Sherman W. Prediction of absolute solvation free energies using molecular dynamics free energy perturbation and the OPLS force field. J Chem Theory Comput. (2010) 6:1509–19. 10.1021/ct900587b 26615687

[52] Martyna GJ Tobias DJ Klein ML. Constant pressure molecular dynamics algorithms. J Chem Phys. (1994) 101:4177–89. 10.1063/1.467468

[53] Kalibaeva G Ferrario M Ciccotti G. Constant pressure-constant temperature molecular dynamics: a correct constrained NPT ensemble using the molecular virial. Mol Phys. (2003) 101:765–78. 10.1080/0026897021000044025

[54] Martyna GJ Klein ML Tuckerman M. Nosé–Hoover chains: the canonical ensemble via continuous dynamics. J Chem Phys. (1992) 97:2635–43. 10.1063/1.463940

[55] Toukmaji AY Board JA. Ewald summation techniques in perspective: a survey. Comput Phys Commun. (1996) 95:73–92. 10.1016/0010-4655(96)00016-1

[56] Kagami LP das Neves GM Timmers LFSM Caceres RA Eifler-Lima VL. Geo-measures: a PyMOL plugin for protein structure ensembles analysis. Comput Biol Chem. (2020) 87:107322. 10.1016/j.compbiolchem.2020.107322 32604028

[57] Rajamanikandan S Jeyakanthan J Srinivasan P. molecular docking, molecular dynamics simulations, computational screening to design quorum sensing inhibitors targeting LuxP of vibrio harveyi and its biological evaluation. Appl Biochem Biotechnol. (2017) 181:192–218. 10.1007/s12010-016-2207-4 27535409

[58] Bharadwaj KK Sarkar T Ghosh A Baishya D Rabha B Panda M. Nature potential for COVID-19: targeting SARS-CoV-2 Mpro inhibitor with bioactive compound. PPR290723. (2021) 193:3371–94. 10.26434/chemrxiv.14112515 PMC8248955 34212286

[59] Piao L Chen Z Li Q Liu R Song W Kong R. molecular dynamics simulations of wild type and mutants of SAPAP in complexed with Shank3. Int J Mol Sci. (2019) 20:224. 10.3390/ijms20010224 30626119 PMC6337207

[60] Panwar U Singh SK. Atom-based 3D-QSAR, molecular docking, DFT, and simulation studies of acylhydrazone, hydrazine, and diazene derivatives as IN-LEDGF/p75 inhibitors. Struct Chem. (2021) 32:337–52. 10.1007/s11224-020-01628-3

[61] Pawara R Ahmad I Surana S Patel H. Computational identification of 2,4-disubstituted amino-pyrimidines as L858R/T790M-EGFR double mutant inhibitors using pharmacophore mapping, molecular docking, binding free energy calculation, DFT study and molecular dynamic simulation. Silico Pharmacol. (2021) 9:54. 10.1007/s40203-021-00113-x 34631361 PMC8494888

[62] Schrödinger. Qikprop 4.4 User Manual. (2015). Available online at: http://gohom.win/ManualHom/Schrodinger/Schrodinger_2015-2_docs/qikprop/qikprop_user_manual.pdf (accessed January 03, 2022).

[63] Schrodinger Release. Schrodinger Release, 2020–2: QikProp, LLC (2020).

[64] Pires DE Blundell T Ascher D. pkCSM: predicting small-molecule pharmacokinetic properties using graph-based signatures. J Med Chem. (2015) 58:4066–72. 10.1021/acs.jmedchem.5b00104 25860834 PMC4434528

[65] Daina A Michielin O Zoete V. SwissADME: a free web tool to evaluate pharmacokinetics, drug-likeness and medicinal chemistry friendliness of small molecules. Sci Rep. (2017) 7:42717. 10.1038/srep42717 28256516 PMC5335600

[66] Abdullahi M Adeniji SE. *In-silico* molecular docking and ADME/pharmacokinetic prediction studies of some novel carboxamide derivatives as anti-tubercular agents. Chem Africa. (2020) 3:989–1000. 10.1007/s42250-020-00162-3

[67] Scafuri B Bontempo P Altucci L De Masi L Facchiano A. Molecular docking simulations on histone deacetylases (HDAC)-1 and−2 to investigate the flavone binding. Biomedicines. (2020) 8:568. 10.3390/biomedicines8120568 33291755 PMC7761979

[68] Bharadwaj S El-Kafrawy SA Alandijany TA Bajrai LH Shah AA Dubey A. Structure-based identification of natural products as SARS-CoV-2 M(pro) antagonist from echinacea angustifolia using computational approaches. Viruses. (2021) 13:305. 10.3390/v13020305 33672054 PMC7919488

[69] Singh R Bhardwaj VK Das P Purohit R. A computational approach for rational discovery of inhibitors for non-structural protein 1 of SARS-CoV-2. Comput Biol Med. (2021) 135:104555. 10.1016/j.compbiomed.2021.104555 34144270 PMC8184359

[70] Domínguez-Villa FX Durán-Iturbide NA Ávila-Zárraga JG. Synthesis, molecular docking, and in silico ADME/Tox profiling studies of new 1-aryl-5-(3-azidopropyl)indol-4-ones: potential inhibitors of SARS CoV-2 main protease. Bioorg Chem. (2021) 106:104497. 10.1016/j.bioorg.2020.104497 33261847 PMC7683933

[71] Laddha AP Murugesan S Kulkarni YA. *In-vivo* and *in-silico* toxicity studies of daidzein: an isoflavone from soy. Drug Chem Toxicol. (2020). 10.1080/01480545.2020.1833906. [Epub ahead of print]. 33059469

[72] Muhammad S Hassan SH Al-Sehemi AG Shakir HA Khan M Irfan M. Exploring the new potential antiviral constituents of Moringa oliefera for SARS-COV-2 pathogenesis: an *in silico* molecular docking and dynamic studies. Chem Phys Lett. (2021) 767:138379. 10.1016/j.cplett.2021.138379 33518774 PMC7835070

[73] Chinnasamy P Arumugam R. *In silico* prediction of anticarcinogenic bioactivities of traditional anti-inflammatory plants used by tribal healers in Sathyamangalam wildlife Sanctuary, India. Egypt J Basic Appl Sci. (2018) 5:265–79. 10.1016/j.ejbas.2018.10.002

[74] Amala M Rajamanikandan S Prabhu D Surekha K Jeyakanthan J. Identification of anti-filarial leads against aspartate semialdehyde dehydrogenase of Wolbachia endosymbiont of *Brugia malayi*: combined molecular docking and molecular dynamics approaches. J Biomol Struct Dyn. (2019) 37:394–410. 10.1080/07391102.2018.1427633 29334340

[75] Khan SA Asiri AM Basisi HM Asad M Zayed MEM Sharma K. Synthesis and evaluation of Quinoline-3-carbonitrile derivatives as potential antibacterial agents. Bioorg Chem. (2019) 88:102968. 10.1016/j.bioorg.2019.102968 31075745

[76] Srivastava R Gupta SK Naaz F Sen Gupta PS Yadav M Singh VK. Alkylated benzimidazoles: design, synthesis, docking, DFT analysis, ADMET property, molecular dynamics and activity against HIV and YFV. Comput Biol Chem. (2020) 89:107400. 10.1016/j.compbiolchem.2020.107400 33068917 PMC7537607

[77] Chinnasamy S Selvaraj G Kaushik AC Kaliamurthi S Chandrabose S Singh SK. Molecular docking and molecular dynamics simulation studies to identify potent AURKA inhibitors: assessing the performance of density functional theory, MM-GBSA and mass action kinetics calculations. J Biomol Struct Dyn. (2020) 38:4325–35. 10.1080/07391102.2019.1674695 31583965

